# Structural Changes of NiFe Layered Double Hydroxides During the Oxygen Evolution Reaction: A Diffraction and Total Scattering *Operando* Study

**DOI:** 10.1002/smll.202411211

**Published:** 2025-02-21

**Authors:** Olivia Aalling‐Frederiksen, Nicolas Schlegel, Stefanie Punke, Andy S. Anker, Gustav K. H. Wiberg, Baiyu Wang, Jens Edelvang‐Pejrup, Freja B. Holde, María Paula Salinas‐Quezada, Nicolas P. L. Magnard, Laura G. Graversen, Matthias Arenz, Rebecca K. Pittkowski, Kirsten M. Ø. Jensen

**Affiliations:** ^1^ Department of Chemistry and Nano‐Science Center University of Copenhagen Copenhagen Denmark; ^2^ Department of Chemistry Biochemistry and Pharmaceutical Sciences University of Bern Bern Switzerland

**Keywords:** layered compounds, nanostructures, operando experiments, oxygen evolution reaction, pair distribution function analysis

## Abstract

NiFe‐layered double hydroxides (LDHs) are promising electrocatalysts for the oxygen evolution reaction (OER) in alkaline media. Here, operando X‐ray diffraction (XRD) and X‐ray total scattering are used with Pair Distribution Function (PDF) analysis to investigate the atomic structure of the catalytically active material and follow structural changes under operating conditions. XRD shows an interlayer contraction under applied oxidative potential, which relates to a transition from the α‐LDH to the γ‐LDH phase. The phase transition is reversible, and the α‐LDH structure is recovered at 1.3 VRHE. However, PDF analysis shows an irreversible increase in the stacking disorder under operating conditions, along with a decrease in the LDH sheet size. The analysis thus shows that the operating conditions induce a breakdown of the particles leading to a decrease in crystallite size.

## Graphical abstract

*Operando* X‐ray diffraction and Pair Distribution Function analysis are used to follow the structural changes taking place in NiFe‐layered double hydroxides when used as electrocatalysts in the Oxygen Evolution Reaction. The analysis shows that the operating conditions induce a breakdown of the particles.

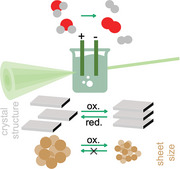

## 1 Introduction

**Figure 1:**
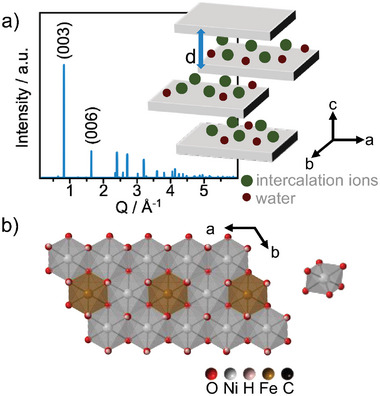
a) Schematic representation of a layered double hydroxide structure with 2D sheets represented as grey slabs, intercalation ions in green, and water in burgundy. The spacing between the layers, *d*‐spacing, is highlighted with a blue arrow. Calculated XRD pattern of an LDH structure. b) Illustration of a sheet with metal oxide [MO_6_] octahedra.

Hydrogen production through water electrolysis (2 H_2_O  →  O_2_ + 2 H_2_) plays a central role in energy conversion and storage for the green transition.^[^ ^1^, ^2^, ^3^ ^]^ Here, a major challenge is oxygen production at the anode, the oxygen evolution reaction (OER), which is a kinetically hindered process requiring large overpotentials.^[^ ^4^, ^5^ ^]^ Therefore, OER catalyst development is key, and there is an especially large interest in developing catalysts using earth‐abundant materials. Transition metal (Mn, Fe, Co, and Ni) oxides and hydroxides are among the most active electrocatalysts in alkaline media.^[^ ^4^, ^6^, ^7^, ^8^ ^]^ For instance, transition metal oxyhydroxides with hydrotalcite‐like layered double hydroxide (LDH) structures, have obtained immense attention over the last decades due to their high OER activity.^[^ ^8^, ^9^, ^10^, ^11^, ^12^, ^13^ ^]^ LDHs are layered materials with 2D sheets built from metal oxide [MO_6_] octahedra (M = Mn, Fe, Co, or Ni). The crystal structure of an LDH, denoted as the α‐LDH, is presented in a simplified way in **Figure** **1**a and the atomic arrangement within the 2D layers is shown in Figure 1b.

In the α‐LDH, the layers are organized in an ABC stacking order. The layers are separated by ions represented by green circles in Figure 1a. The intercalation ions provide charge compensation in the layered material, and are commonly small anions such as CO_3_^2−^,^[^ ^14^ ^]^ Cl^−^,^[^ ^15^ ^]^ ClO_4_^−^,^[^ ^16^ ^]^ or SO_4_^2−^,^[^ ^17^ ^]^ but can also be larger organic species.^[^ ^18^ ^]^ Additionally, a γ‐LDH with a AAA stacking order is known. Here, cations such as K^+^ or Na^+^, are instead intercalated.^[^ ^19^, ^20^ ^]^ Water is furthermore intercalated in between the layers in both the γ‐ and the α‐phase.^[^ ^4^, ^11^ ^]^ The nature of the intercalating ions has a strong influence on the layer spacing.^[^ ^8^ ^]^ α‐LDH with CO_3_^2−^ ions intercalated have layer spacing of 7.8 Å, while the γ‐LDH with K^+^ ions has a layer spacing of 7.1 Å.^[^ ^21^ ^]^

The calculated XRD pattern of a LDH structure is shown in Figure 1a. The XRD peaks observed at low *Q*‐values are directly related to the interlayer spacing in the structure and can be assigned to and indices if considering a structure where one unit cell contains three layers. These characteristic peaks thus provide direct information on the layer spacing. However, the atomic arrangement within the layers can be more challenging to resolve from conventional XRD analysis, as a large degree of stacking disorder is often present in this type of material. Here, characterization methods such as Extended X‐ray Absorption Fine Structure (EXAFS)^[^ ^20^, ^22^ ^]^ and PDF^[^ ^23^ ^]^ analysis of X‐ray total scattering data can provide complementary information to obtain a more complete understanding of the local and intermediate‐range atomic structure.

Within the layered structure type family, the NiFe‐LDH is the most active electrocatalyst for OER.^[^ ^5^, ^8^, ^20^, ^24^ ^]^ The potential of NiFe‐LDH has thus led to extensive studies of the OER performance,^[^ ^25^, ^26^, ^27^ ^]^ and the activity‐promoting effects of Fe have long been, and are still, studied and debated.^[^ ^28^, ^29^, ^30^, ^31^ ^]^ Structural characteristics such as site defects or the degree of crystallinity have been shown to significantly influence the electrocatalytic activity.^[^ ^32^, ^33^, ^34^, ^35^, ^36^ ^]^ Resolving the effect of these various structural features on the activity is challenging, and the complexity increases as structural changes are known to take place in the catalyst material under reaction conditions,^[^ ^37^, ^38^ ^]^ and especially surface restructuring or amorphization has been suggested.^[^ ^39^, ^40^, ^41^, ^42^ ^]^ In the case of LDH‐structured catalysts, it has furthermore been shown how NiFe‐ and CoFe‐LDHs transform from the as‐prepared α‐phases to deprotonated catalytically active γ‐phases.^[^ ^21^ ^]^ This has been resolved through *operando* experiments for NiFe‐LDH catalysts during electrocatalysis:^[^ ^20^, ^21^, ^43^, ^44^, ^45^ ^]^ While XRD studies have shown this contraction in the layer‐layer distance under OER conditions,^[^ ^21^ ^]^ EXAFS studies have provided insights on the local structural changes showing contractions in the M‐O and M‐M (M = metal) distances.^[^ ^20^, ^45^ ^]^

The active site and oxygen evolution mechanism is still not fully elucidated. Indications have been found for both Ni^[^ ^46^, ^47^, ^48^ ^]^ and Fe^[^ ^20^, ^31^, ^49^ ^]^ being catalytically active, while recent studies find that a dynamic Fe active site is likely to evolve the oxygen.^[^ ^50^, ^51^ ^]^ Importantly, very little is known about the structural changes in the catalytically active layers, i.e., the intermediate range atomic orderas well as the occurrence of structural disorder, under *operando* conditions. Here, analysis of PDFs, obtained from X‐ray total scattering data, is well‐suited. In a recent study, we showed how *operando* PDF can provide insights into ultra‐small Ir nanoparticles during acidic OER, and revealed the formation of a highly disordered oxide.^[^ ^52^ ^]^

Here, we investigate NiFe‐LDH electrocatalysts under alkaline OER conditions by combining *operando* X‐ray diffraction and total scattering with PDF analysis. We use a custom‐made set‐up that allows performing such scattering experiments under OER conditions.^[^ ^53^ ^]^ Firstly, our data confirm that the material undergoes a phase transition with a contraction in the layer‐spacing (α‐to‐γ phase transition), suggesting that γ‐LDH is the active phase. The phase transition is reversible, and the α‐LDH structure is recovered at 1.3 V_RHE_. However, our analysis shows an irreversible increase in the layer stacking disorder under operating conditions. We also observe an irreversible decrease in the sheet size. The *operando* experiments thus show that the OER conditions lead to a breakdown of the as‐synthesized crystallites, both in terms of increased stacking disorder and smaller sheets.

## 2 Results and Discussion

### 2.1 Characterization of the As‐Prepared Material

The NiFe‐LDH powders used for our *operando* experiments were prepared through a simple hydrothermal synthesis^[^ ^54^ ^]^ The Ni:Fe ratio and the synthesis time at 140 °C were varied, and we study three samples with 4:1 Ni:Fe at 30 h (Ni4Fe:30h), 4:1 Ni:Fe at 2 h (Ni4Fe:2h) and 1:0 Ni:Fe at 2 h (Ni:2h). Before discussing the *operando* experiments, we consider the structural characteristics of the prepared catalyst material. We furthermore use data from the as‐synthesized particles to develop a strategy for structure characterization that will be used for the *operando* data of these complex materials. We initiate our analysis by focusing on the Ni4Fe:30h catalyst and return to the other samples below.

**Figure 2:**
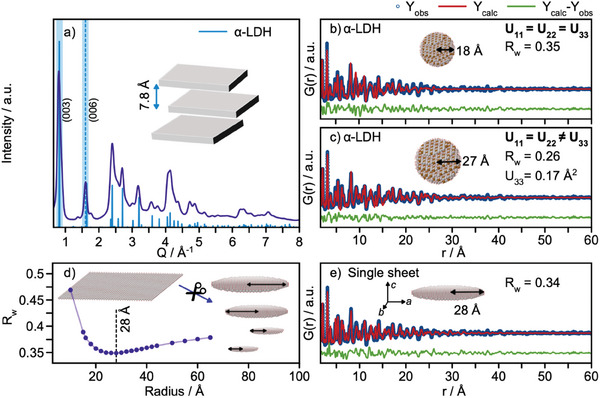
a) X‐ray total scattering data collected for the as‐prepared Ni4Fe:30h (purple). The calculated pattern of α‐LDH is plotted in blue. PDF refinements using the α‐LDH as the structural model with b) isotropic ADP‐values and c) anisotropic ADP‐values. d) radius versus R_w_ and a demonstration of the sheet‐cutting procedure, where a single sheet is extracted from the α‐LDH structure. e) PDF refinement using a sheet with a 28 Å radius.

The scattering pattern from this sample is shown in **Figure** **2**a, where the reflections are highlighted. These two reflections are directly related to the characteristic interlayer distance (*d* = 7.8 Å) of the α‐LDH crystal structure. This interlayer distance is typical for carbonate ions inside the LDH structure,^[^ ^55^, ^56^ ^]^ which are produced from urea in the hydrothermal synthesis. A fraction of nitrate ions as remnants from the nitrate precursors could also be present inside the layers of the material. The reflections at higher *Q*‐values (> 2 Å^−1^) agree well with the calculated pattern from α‐LDH.^[^ ^21^ ^]^ However, these are broadened and asymmetrical, indicating a high degree of stacking disorder. Figure S2 (Supporting Information) shows TEM images from samples prepared through the same synthesis route.^[^ ^57^ ^]^ These show that the particles are polydisperse and anisotropic, meaning that larger sheet‐like as well as smaller spherical particles form, which might also affect the peak shape in the scattering data.

For structural modelling, we turn to analysis of the PDF, which is obtained from X‐ray total scattering data. We first use a model based on the crystal structure of NiFe α‐LDH,^[^ ^21^ ^]^ with the fit shown in Figure 2b. Refined parameters are reported in Table S1 (Supporting Information). We refine the scale factor, the lattice parameters (*a*, *b*, and *c*), isotropic atomic displacement parameters (ADPs) of Ni and O within the layers, a δ_2_ parameter accounting for correlated atomic motions and a sp‐diameter, accounting for the finite crystallite size. As seen from the difference curve in Figure 2b, the model gives a relatively poor fit to the intensity and width of the PDF peaks. We expect this to be due to stacking disorder. We therefore attempted a different fit strategy, where the fit was done using anisotropic ADPs $U_{11}=U_{22}\neq U_{33}$ for the metal sites in the LDH structure (Figure 2c). For layered materials, anisotropic ADP refinements can be used as a proxy for turbostratic layer disorder, as previously done for, e.g., CdSe,^[^ ^58^ ^]^ and stacked organic polymers.^[^ ^59^ ^]^ Using anisotropic ADPs improves the fit quality, and our refinement shows that the U_33_ value is relatively large, here 0.17 Å^2^ compared to U_11_ and U_22_ (0.004 Å^2^) which is a clear indication of stacking disorder. The refinements show an inter‐layer spacing of 7.8 Å and a crystallite size radius of ca. 27 Å. We note here that the simple model used assumes a spherical particle. As seen from our TEM micrographs in Figure S2 (Supporting Information), the particles are highly anisotropic, and the refined crystallite size should thus be interpreted as a rough estimate.

To further explore the effect of stacking disorder, and to provide an estimate of the size of the particles in the *ab* plane, i.e., the sheet size, we use another refinement approach, where the model is a single sheet of edge‐sharing [MO_6_] octahedra extracted from the α‐LDH crystal structure. A similar approach has previously been used for the characterization of NiFe‐LDH^[^ ^23^ ^]^ and cobalt oxide.^[^ ^60^, ^61^, ^62^ ^]^ To find the best sheet model to describe our data, we built one large single sheet of the LDH consisting of only metal and oxygen atoms in an octahedral geometry ([MO_6_] octahedra). The sheet has a height of 1.98 Å and consists of 19 999 metal atoms, from which we cut different sheet sizes in the shape of disks ranging from 10 to 65 Å in radius (38 to 1592 metal atoms) as illustrated in Figure 2d. For each sheet model, a PDF refinement was performed, where the scale factor was refined together with three parameters (*a*, *b*, and *c*) which allows a contraction and/or expansion of the discrete model.^[^ ^63^ ^]^ The isotropic ADP of Ni was furthermore refined, while the isotropic ADP of O was fixed to 0.003 Å^2^. The agreement factors (R_w_‐values) are plotted as a function of the sheet radius in Figure 2d. A sheet with a radius of 28 Å results in the best description indicated by the lowest R_w_‐value (0.35). We note that all sheet radii between 20 and 40 Å give similar R_w_‐values which might indicate a large sheet size distribution, as also expected from the TEM micrographs presented in Figure S2 (Supporting Information). The fit with radius 28 Å is presented in Figure 2e, while the refined parameters are listed in Table S2 (Supporting Information). While the sheet model does not account for all intensities in the experimental data, the model describes most of the PDF features well, indicating that intralayer distances give rise to the most dominating intensities in the experimental PDF. This again confirms that the stacking disorder is significant. To further validate these results, the experimental PDF is compared to the calculated PDFs of both the α‐LDH crystal structure and a single sheet extracted from the layered structure in Figure S3 (Supporting Information). In the *r*‐range shown (4 – 14 Å), interlayer atom‐atom correlations should be present, as highlighted by the colored lines in the figure. However, the peak intensities of two distinct interlayer correlations (10 and 11.9 Å) are lowered for the experimental data, which points toward this dominance of the intralayer features in the experimental PDF.

In summary, our characterization of the structure of the as‐synthesized Ni4Fe:30h catalyst shows that it takes the α‐LDH structure, but with a high degree of stacking disorder. At the same time, we see that while XRD analysis gives direct, easily accessible information on the layer stacking (from the presence of (003) and (006) Bragg peaks), the PDF can be used for refinement of the interlayer structure and can give an estimate of the sheet size. We will apply these methodologies for structural studies under operating conditions.

### 2.2 Operando XRD

**Figure 3:**
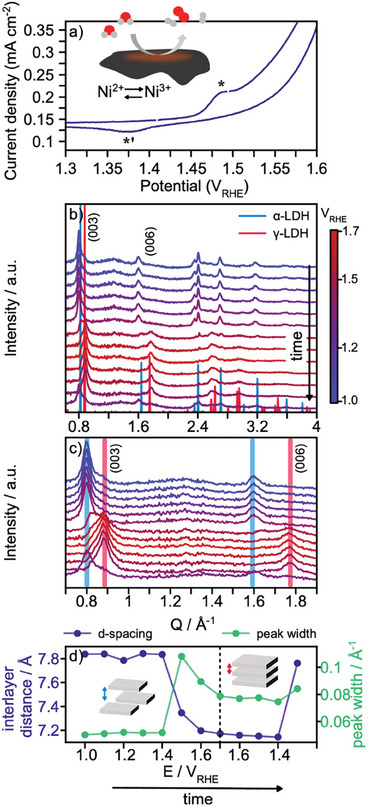
a) Excerpt of a cyclic voltammogram of Ni4Fe:30h recorded in 0.1 M KOH electrolyte. * and *’ indicate the redox features. b) *Operando* XRD data for Ni4Fe:30h. For each potential hold, an XRD pattern is collected when a *quasi*‐steady state is reached after 2 min. Calculated XRD patterns of the a α‐ and a γ‐LDH are also plotted.^[^ ^21^ ^]^ c) XRD patterns presented in b) in the low *Q‐*range (0.7 to 1.9 Å^−1^). e) Interlayer distance and (003) peak width (FWHM) as a function of applied potential.

We now investigate how alkaline OER conditions impact the structure of the catalyst material. Again, we focus on the Ni4Fe:30h sample. The redox chemistry is first studied and **Figure** **3**a shows the corresponding cyclic voltammogram (CV) recorded ex situ. Here, we observe a redox transition with an oxidation peak at 1.49 V_RHE_(*) and a related reduction peak in the negative‐going scan at 1.38 V_RHE_(*’). These redox peaks are most likely associated with Ni oxidation and agree with observations on similar materials.^[^ ^21^, ^64^ ^]^ The redox transition appears to overlap with the onset of the OER.^[^ ^21^ ^]^

Using *operando* XRD, we first investigate the changes in interlayer spacing in the potential range of 1.0 to 1.7 V_RHE_. The three‐electrode flow set‐up used for our *operando* experiments is described by Wiberg et al.^[^ ^53^ ^]^ From the currents collected during the *operando* XRD experiment, we observe that the potential of the OER onset is ≈1.5 V_RHE_ which agrees with the CV presented in Figure 3a. The electrochemical data from the chronoamperometry is shown in Figure S4 (Supporting Information). At 1.6 V_RHE_, we reach a steady‐state current density of circa 10 mA cm^−2^ in the *operando* electrochemical cell.

Figure 3b shows the XRD patterns collected. We increase the potential in potential steps of 0.1 V to 1.7 V_RHE_, followed by a potential step protocol in the reverse direction. For each potential hold, an XRD pattern is collected when a *quasi*‐steady state is reached after 2 min.

We first focus on the evolution of the (003) and (006) reflections, highlighted in Figure 3c, which shows a zoom‐in of Figure 3b to emphasize the *Q‐*range from 0.7 to 1.9 Å^−1^. The evolution of the interlayer spacing, as determined from the position of the (003) peak (Figure S5, Supporting Information), is shown in Figure 3d. A contraction of the interlayer distance is observed from 7.8 Å for the as‐prepared catalyst to 7.1 Å at a potential of 1.5 V_RHE_, which correlates with the potential of the Ni oxidation. The resulting phase appears to be the catalytically active material in OER, as no further shift in the interlayer distance is observed at the potentials where significant OER currents are measured, i.e.10 mA cm^−2^ and 20 mA cm^−2^ at 1.6 and 1.7 V_RHE_, respectively. This α‐to‐γ phase transition agrees with the Bode diagram, which describes interlayer spacing variations in Ni‐hydroxides based on applied potential and the reaction from Ni(OH)_2_ to NiOOH.^[^ ^65^ ^]^ A similar observation was recently made by Dionigi et al.^[^ ^21^ ^]^ During the α‐to‐γ phase transition, Bragg‐peak broadening is also detected. We highlight this trend in Figure 3d, which shows the evolution of (003) peak width, estimated by fitting a simple Gaussian function. This indicates crystallite size changes and/or an increased disorder along the stacking direction. The peak width is converted to crystallite size in Figure S6 (Supporting Information) using the Scherrer equation. Here, all broadening is assumed to be size‐related, and we observe a clear decrease in crystallite size at potentials above 1.5 V_RHE_.

A γ‐to‐α phase transition is observed when following the potential step protocol in the backward direction to 1.3 V_RHE_, Figure 3b,c. This reversibility agrees with other studies on similar systems.^[^ ^21^, ^65^ ^]^ However, the width of the (003) peak continues to broaden indicating that the decrease in crystallinity or increase in disorder in the stacking direction is irreversible (Figure 3d).

### 2.3 Operando PDF

While structural information on the interlayer correlations is obtained from XRD, insights into the intralayer atom‐atom correlations are more accessible from PDF analysis. *Operando* X‐ray total scattering data for PDF analysis were thus collected. The electrochemical *operando* cell used in the X‐ray scattering experiments is shown in Figure S7 (Supporting Information).^[^ ^66^ ^]^ To ensure that the data collected in XRD and PDF experiments are comparable, we compare the electrochemical response in the two experiments in Figure S8 (Supporting Information).

**Figure 4:**
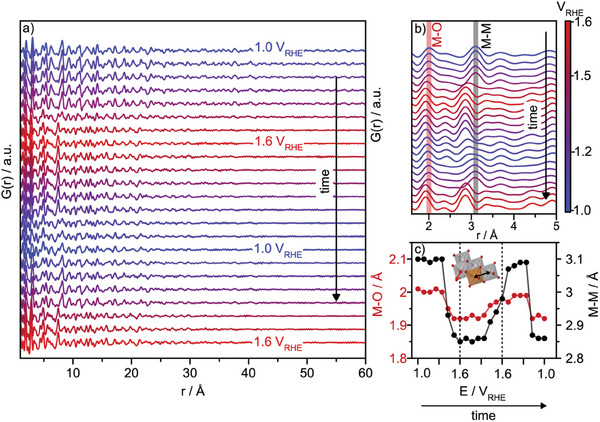
a) *Operando* PDFs for Ni4Fe:30h. b) The local *r*‐range (1.6 – 4.0 Å) of the *operando* PDFs shown in a). c) *r*‐positions of the closest M‐O and M‐M PDF peak for each potential step. A cut‐out from the LDH structure is shown, where the two atom‐atom distances are highlighted with arrows.

**Figure** **4**a shows the PDFs acquired at each potential step. Here, the step protocol is repeated twice to investigate the reversibility of the phase transition (1.0 V_RHE_ to 1.6 V_RHE_ to 1.0 V_RHE_ to 1.6 V_RHE_, with steps of 0.1 V).

We first consider the data and structural changes qualitatively. Upon applying more positive potentials of >1.0 V_RHE_ a change in the PDF dampening is observed, Figure 4a. The PDF collected at an applied potential of 1.6 V_RHE_ terminates at a lower *r*‐value than for the as‐prepared material. Four PDFs at four stages of the reaction (1.0 V_RHE_ (before OER), 1.6 V_RHE_ (first), 1.1 V_RHE_ (after OER) and 1.6 V_RHE_ (second)) are presented and directly compared in Figure S9 (Supporting Information) to highlight this change. This dampening most likely relates to a decrease in crystallite size or an increased disorder/surface amorphization of the in‐plane structure at increased potential, as will be discussed further below.^[^ ^67^, ^68^ ^]^ When focusing on the very local *r*‐range (1.6 – 4.0 Å) in Figure 4b, we observe a PDF‐peak shift toward lower *r*‐values as the applied potential reaches 1.5 V_RHE_. This is highlighted in Figure 4c, showing the evolution of the first M‐O and M‐M distances. This observation relates to the α‐to‐γ phase transition we followed with *operando* XRD and agrees with the oxidation of Ni and the phase transition from Ni(OH)_2_ to NiOOH.^[^ ^20^, ^45^, ^69^ ^]^ When decreasing the potential back to 1.0 V_RHE_, a slow recovery of the local structure of the initial material structure is observed. In the local range, there seems to be an almost complete recovery of the catalyst material. However, comparing the PDFs collected at 1.0 V_RHE_ before OER, and at 1.1 V_RHE_ after OER (Figure S9, Supporting Information) there are inconsistencies at the higher *r*‐values.

**Figure 5:**
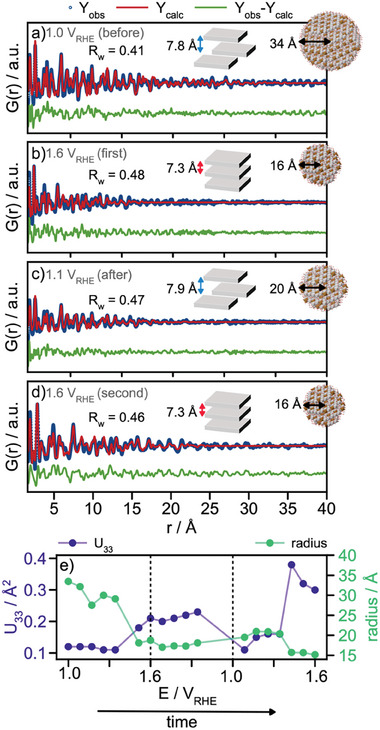
Refinements for Ni4Fe:30h operando PDFs for a) 1.0 V_RHE_, b) 1.6 V_RHE_, c) 1.1 V_RHE_ and d) 1.6 V_RHE_. e) Refined spherical crystallite size and the anisotropic ADP value U_33_ as a function of the applied potential.

For more quantitative information, we turn to PDF modeling. We use the crystalline α‐LDH structure as the starting model for the PDFs obtained at 1.1 V_RHE,_ while the γ‐phase is used for the 1.6 V_RHE_ data. **Figure** **5**a–d show exemplary fits to the PDFs collected at four different potential steps (1.0 V_RHE_ (before OER), 1.6 V_RHE_ (first), 1.1 V_RHE_ (after OER), and 1.6V_RHE_ (second)). Here, we refine the scale factor, the lattice parameters, the δ_2_ parameter, the anisotropic ADP‐values, and the sp‐diameter. All refined parameters for the four stages of the electrochemical protocol are listed in Table S3 (Supporting Information). From these refinements, we first observe a contraction in the layer distance when going from 1.0 V_RHE_ to 1.6 V_RHE_ (from *d* = 7.8 Å to *d* = 7.4 Å). This was already elucidated from our XRD analysis, Figure 3b. We note that a difference is observed between the refined c‐values from PDF and XRD, although the trends are similar. Generally, the lower *Q‐*resolution in total scattering measurements compared to XRD means that lattice parameters can be more precisely determined from XRD than PDF.

The refined U_33_ parameters are plotted as a function of applied potential in Figure 5e. While already enlarged before OER conditions (0.1 Å^2^), the U_33_‐value increases even further with a potential of 1.5 V_RHE_ or higher and changes drastically during the second cycle, where it refines to 0.3 Å^2^, indicating a very large degree of stacking disorder.

From the PDF analysis, we also obtain information about how the crystallite size changes under OER conditions from the PDF analysis. Figure 5e shows the crystallite radius as a function of applied potential (extracted from the refined sp‐diameter). The spherical crystallite radius is initially refined to 34 Å for the PDF collected at 1.0 V_RHE_, Figure 5a. We note that this stage of the reaction should be comparable to the as‐synthesized sample, where the size was found to be 27 Å. We assign this small difference to slightly different instrument resolutions for the two measurements. As the potential is increased to the first potential step at 1.6 V_RHE_ during the first ramp, we see a clear decrease in size, which refines to 16 Å, see Figure 5b,e. The crystallite size is not fully recovered as the potential is stepped to 1.1 V_RHE_ (20 Å) and again to 1.6 V_RHE_ (16 Å).

**Figure 6:**
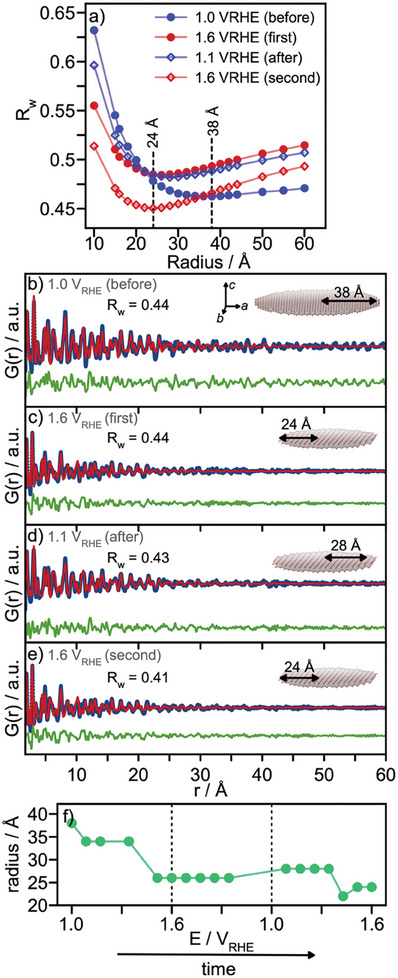
a) R_w_ value versus sheet radius for the four different stages of the reaction. b‐e) Exemplary PDF refinements with single sheet models of the optimal sheet sizes for four different potentials. f) Optimized Sheet radius as a function of the potential applied during the measurement.

To further separate the contributions from the inter‐ and intralayer structure in the PDF, we again use the sheet‐cluster approach presented for the as‐synthesized material in Figure 2. **Figure** **6**a shows the fit R_w_ plotted against the sheet radius for four stages of reaction. These refinements (Figure 6b–e, Table S4, Supporting Information) show a clear decrease in sheet size with applied potential, which is not recovered. The sizes extracted from every potential step in the *operando* total scattering experiments are presented in Figure 6f, illustrating the irreversible nature of the size reduction over the complete potential range.

Our combined *operando* XRD and PDF analyses thus provide information on both the changes in inter‐ and intralayer structure of the LDH under reaction conditions. Firstly, we confirm that the catalytically active phase at the OER potential is the γ‐LDH structure. This finding agrees with literature reports of the γ‐phase being the stable phase, which is present under the oxidative OER conditions.^[^ ^21^, ^70^ ^]^ When reaching this phase under operating conditions, the XRD clearly shows that the layer stacking becomes further disordered, and the coherent domain size decreases. From the PDF, we furthermore observe a decrease in the sheet size, which is irreversible. Previous studies have suggested the formation of amorphous LDH surfaces on transition metal oxides during operation.^[^ ^38^, ^42^ ^]^ Our data illustrate that nanoscale order is preserved in the particles, and full amorphization does not take place. Surface amorphization could be responsible for the decrease in refined crystallite and/or sheet size. However, in PDF analysis, the formation of a significant amorphous fraction can often be observed by an increase in the intensity of the peaks in the low *r‐*range at the expense of long‐range order peaks.^[^ ^71^ ^]^ We do not observe this effect in our data, which suggests that the largest effect is breaking down the LDH crystallites into smaller particles, and not surface amorphization. SAXS data, also collected during *operando* conditions corroborate this hypothesis. The SAXS data are shown in Figures S10‐S11 (Supporting Information). As the particles are agglomerated, polydisperse and anisotropic, detailed SAXS analyses are not possible. However, we can still observe a decrease in mean particle radius from 46 to 39 Å with increased applied potential going from 1.0 to 1.7 V_RHE_ (Table S5, Supporting Information).

### 2.4 Operando XRD and PDF on Additional Samples

**Figure 7:**
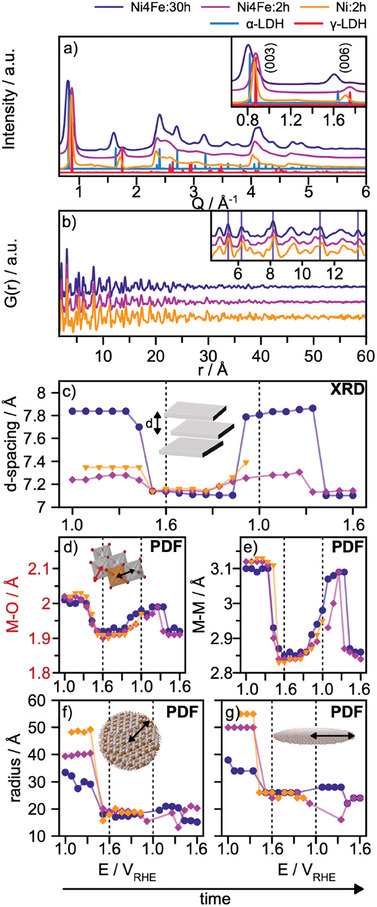
a) Total scattering data collected for the three samples; Ni4Fe:30h, Ni4Fe:2h, and Ni:2h. We note that the data for the two Ni4Fe catalysts (30h and 2h) were collected in an ex situ capillary set‐up at the P02.1 beamline, DESY, while the data for the Fe‐free catalyst (Ni:2h) was collected from the sample in the *operando* set‐up at DanMAX, MAXIV. b) PDFs obtained for the three samples. c) d‐spacing as a function of applied potential. d) *r*‐positions of the closest M‐O peak for and e) *r*‐positions of the closest M‐M peak. f) Crystallite radius extracted from PDF refinements using the α‐LDH crystal structure and g) Crystallite radius extracted from PDF refinements using a single sheet model.

Having established the behavior of the Ni4Fe:30h catalyst, we now return to the additional samples to map the influence of the initial structure of the material on the changes taking place under operating conditions. In addition to Ni4Fe:30h, we prepared two other samples: Ni4Fe:2h and Ni:2h, i.e., varying synthesis time and Fe content. We have previously demonstrated how synthesis time leads to larger *d*‐spacing of as‐prepared NiFe‐LDH powders.^[^ ^57^ ^]^ This is confirmed in **Figure** **7**a, showing scattering data for the as‐prepared materials. As already presented in the analysis above, we extract a *d*‐spacing of 7.8 Å for the as‐prepared Ni4Fe:30h catalyst. A *d*‐spacing of ≈ 7.2 Å is obtained for both Ni4Fe:2h and Ni:2h catalysts. Hence, the Ni:Fe atomic ratio appears to have a smaller effect on the material structure than synthesis time. Figure 7b shows PDFs for the three samples, with PDF fits in Figure S12 (Supporting Information) and Tables S6,S7 (Supporting Information). Comparing first the Fe‐containing samples, Ni4Fe:30h and Ni4Fe:2h, with each other, we find a slightly larger refined crystallite sheet size for the 2h (34 Å) compared to the 30h sample (28 Å), which is surprising, since longer heating time often leads to an increased crystallite size. However, we also extract a larger anisotropic ADP value when using the α‐LDH structural model, 1.0 Å^2^ for the 2h compared to the 0.20 Å^2^ for the 30h sample, which suggests that the 2h sample contains more stacking disorder. This observation agrees with the high Q features in the *Q*‐space data in Figure 7a, which are broader and more asymmetric for the 2h sample, compared to the 30h sample. The analysis for the Fe‐free sample (Figure S13e–h, Supporting Information) shows that 45 Å crystallites form with a very high degree of stacking disorder (U_33_ = 2.8 Å^2^). Our analysis thus shows that the three samples are structurally comparable, but that the stacking distance and degree of disorder varies for the two 2h samples compared to the 30h sample.

We now investigate the structural changes occurring during OER through *operando* total scattering studies. All *operando Q‐*space data along with the corresponding PDFs for the three experiments are shown in Figure S13 (Supporting Information) and the recorded OER current densities in Figure S14 (Supporting Information), while the analysis results are compared in Figure 7. We note that for the Fe‐free catalyst (Ni:2h) the potential step protocol was only followed from 1.1 V_RHE_ to 1.6 V_RHE_ and back to 1.0 V_RHE_, whereas for the two 2h samples 1.6 V_RHE_ was reached in a second cycle. We first focus on the interlayer spacing, obtained from the *Q‐*space data, Figure 7c. Here, the *d*‐spacing is obtained from the *Q‐*space data collected during the total scattering experiments and extracted from the position of the (006) reflection, as part of the (003) reflection is cut off by the position of the beam stop.

As clearly seen from the *Q‐*space data (Figure S12, Supporting Information) and the extracted *d*‐spacing (Figure 7c), we again observe a transition from the α‐ to the γ‐LDH phase. As expected from the analysis of the as‐prepared samples above, we observe a large difference in the starting values of the interlayer spacing. However, when getting to OER conditions, we observe how the initial layer spacing does not appear to significantly influence the layer spacing of the catalytically active phase. The analysis provided in Figure 7c shows that all three samples transform into the γ‐LDH with a *d*‐spacing of ≈ 7.1 Å at an applied potential of 1.6 V_RHE_, independently of the spacing of the as‐prepared material. We further follow the changes in the closest M‐O and M‐M atom‐atom distances extracted from the collected *operando* PDFs, Figure 7d,e, respectively. Here, we observe that the very local structural environment is similar for the three as‐prepared samples, and they all experience a similar contraction with a decrease in the two atom‐atom distances with oxidation.

Lastly, we follow the changes in the crystallite using the PDF refinement approaches described above, where the crystal structure of the α‐LDH (Figure 7f) and a single sheet extracted from the α‐LDH (Figure 7g) are used as the structural starting models. Just like the layer‐spacing and local structure, the refined size of the three at 1.5 V_RHE_ are very similar, even if they start at different sizes. Regardless of the initial crystallite size, a radius of circa 20 Å is obtained for all three samples under OER conditions, and the size is not recovered in the backward potential steps. Again, we do not observe any indication of amorphization in the data and suggest that the changes are related to a breakdown of the crystallites into smaller particles.

Our results show that, considering the structural characteristics evaluated here, the nature of the as‐synthesized materials does not influence the structure of the active material. It further provides a possible explanation for our recent finding, where two as‐prepared NiFe‐LDH materials with different crystallite sizes and *d*‐spacings are performing equally well catalytically.^[^ ^57^ ^]^

## 3 Conclusion

We have used *operando* XRD and total scattering with PDF analysis to elucidate the structural changes of α‐NiFe‐LDH during OER. The combination of diffraction and total scattering is useful for obtaining in‐depth knowledge of both inter‐ and intralayer structural changes of the catalyst materials. We first observe a decrease in the interlayer distance when going to oxidative potentials (1.6 V_RHE_). This decrease relates to the transition to the catalytically active γ‐LDH phase. This change in structure is likely related to an exchange of intercalation ions, i.e., carbonate for potassium.^[^ ^21^, ^72^, ^73^ ^]^ The phase transition is reversible, and the α‐LDH structure is recovered at 1.3 V_RHE_ of the backward direction of the potential step protocol. However, our analysis shows an irreversible increase in the stacking disorder under operating conditions. The LDH sheet size is also observed to decrease to circa 20 Å in radius, irrespective of the initial crystallite size. The *operando* experiments show that the OER conditions lead to a breakdown of the as‐synthesized crystallites, both in terms of increased stacking disorder and smaller sheets, hence not a material amorphization. Finally, we show that the size and interlayer spacing of different as‐synthesized NiFe‐LDH materials do not appear to influence the structure of the catalytically active phase.

## Conflict of Interest

The authors declare no conflict of interest.

## Author Contributions

O.A., R.P., M.A., and K.M.Ø.J. planned the project. O.A. collected the total scattering data, did the analysis of the diffraction and total scattering data, and wrote the original draft along with K.M.Ø.J. R.K.P. helped with the experimental planning, data collection and data analysis. G.K.H.W., A.S.A., N.S., B.W., J.E.P., F.B.H., MPSQ, NPL.M., L.G.G., and S.P. took part in collecting the *operando* scattering data. A.S.A., N.S., and S.P. took part in analyzing the data. M.A. and K.M.Ø.J. supervised the project. All authors contributed to reviewing and editing of the paper.

## Supporting information



Supporting Information

## Data Availability

The data that support the findings of this study are available from the corresponding author upon reasonable request.
