# Supplementary material for: Structural Changes of NiFe Layered Double Hydroxides During the Oxygen Evolution Reaction: A Diffraction and Total Scattering Operando Study
Source: Small. 2025 Feb 21;21(12):2411211. doi: 10.1002/smll.202411211 (PMC11947514; doi:10.1002/smll.202411211)
Supplement: Supplementary file 1 — Supporting Information [file SMLL-21-2411211-s001.pdf]

NANO · MICRO
small

Supporting Information

for *Small*, DOI 10.1002/smll.202411211

Structural Changes of NiFe Layered Double Hydroxides During the Oxygen Evolution Reaction: A Diffraction and Total Scattering *Operando* Study

Olivia Aalling-Frederiksen, Nicolas Schlegel, Stefanie Punke, Andy S. Anker, Gustav K. H. Wiberg, Baiyu Wang, Jens Edelvang-Pejrup, Freja B. Holde, María Paula Salinas-Quezada, Nicolas P. L. Magnard, Laura G. Graversen, Matthias Arenz, Rebecca K. Pittkowski* and Kirsten M. Ø. Jensen**

Supporting Information for

Structural changes of NiFe layered double hydroxides during the oxygen evolution reaction: a diffraction and total scattering *operando* study

Olivia Aalling-Frederiksen[1], Nicolas Schlegel [1,2], Stefanie Punke[1], Andy S. Anker[1], Gustav K. H. Wiberg[2], Baiyu Wang[1], Jens Edelvang-Pejrup[1], Freja B. Holde[1], María Paula Salinas-Quezada[1], Nicolas P. L. Magnard[1], Laura G. Graversen[1], Matthias Arenz[2], Rebecca K. Pittkowski*[1] and Kirsten M. Ø. Jensen[1]**

1: Department of Chemistry and Nano-Science Center, University of Copenhagen, Denmark
2: Department of Chemistry, Biochemistry and Pharmaceutical Sciences, University of Bern, Switzerland

Corresponding authors: kirsten@chem.ku.dk, rebecca.pittkowski@chem.ku.dk, matthias.arenz@unibe.ch

Experimental Methods

Catalyst Synthesis

NiFe-LDH nanoparticles were synthesized using a similar approach to the one published by Lu et al.[1] and also demonstrated by Wang and Schlegel et al.[2] $Fe(NO_3)_3{\cdot}9H_2O$ (Sigma-Aldrich, ACS Reagent, ≥98%) and $Ni(NO_3)_2{\cdot}6H_2O$ (Sigma-Aldrich, purum p.a., crystallized, ≥97.0%) were used as precursors with a total concentration of 0.028 M in water. Urea ($CO(NH_2)_2$) (Alfa Aesar, ACS, 99.0–100.5%) was added (0.16 M) and as a hydrolysis agent. The solutions were heated in Teflon-lined steel autoclaves at 140 °C for 2 or 30 h. The reaction mixtures were cooled, and the precipitates were washed and centrifuged in ethanol three times at 4500 rpm. The powders were dried overnight for further characterization. Three samples were prepared by varying the Ni:Fe ratio and the synthesis time at 140 °C: 4:1 Ni:Fe at 30 h (Ni4Fe:30h), 4:1 Ni:Fe at 2 h (Ni4Fe:2h), and 1:0 Ni:Fe at 2 h (Ni:2h). The Ni:Fe ratio used in the hydrothermal synthesis is maintained in the obtained product powders.[2]

Ex situ total scattering

Ex situ total scattering data were collected for the two samples 4:1 Ni:Fe (30h@140°C) and 4:1 Ni:Fe (2h@140°C) at the P02.1 beamline at the Deutsches Elektronen-Synchroton (DESY), Hamburg, Germany. The powder samples were loaded in 1 mm Kapton capillaries and a Varex XRD 4343CT with 150×150 μm^2 pixel size, 2880×2880 pixel area was used to collect 2D data which was subsequently azimuthally integrated with the PyFAI software.[3] A sample-to-detector distance of 300.5 mm was used to cover a large *Q*-range resulting in a $Q_{maxinst}$ = 20.0 $Å^{-1}$, Q_{max} = 20.0 $Å^{-1}$, and Q_{min} = 0.65 $Å^{-1}$. The X-ray energy was 60 keV corresponding to an X-ray wavelength of λ = 0.207 Å. The total scattering data were further treated to obtain the PDFs using the PDFgetX3 software.[4] The PDF modelling was performed using the PDFgui[5] and DiffPy-CMI programs.[6]

Operando GDE scattering experiments

The catalyst inks for all *operando* scattering experiments were prepared by taking 4 mg catalyst and adding it to a solution of ultrapure 1 mL H_2O and 3 mL isopropyl alcohol. 2.6 μL Nafion™ solution (Sigma Aldrich, ~5% in a mixture of aliphatic alcohols and water) was added to the suspension. The 4 mL solution was diluted 10 times and vacuum filtrated onto a gas diffusion layer (GDL) coated with a C-based microporous layer (MPL) (Freudenberg H23C8, Fuel Cell Store). Hereby we obtained a nominal loading of 400 μg cm^{-2} on the gas diffusion electrode (GDE). We used an electrochemical cell described in detail by Wiberg et al.[7] The electrochemical cell is made from polysulfone (PSU) which is resistant to the alkaline electrolyte and provides good X-ray transparency. The GDE functions as the working electrode (WE) and a Ni wire as the counter electrode (CE). As ra eference, an Ag/AgCl (ET072-1 Leakless, eDAQ) for experiments performed at ID31 at European Synchrotron Radiation Facility (ESRF), while a trapped hydrogen electrode (RHE) was used at DanMAX, MAXIV.[8] The solution resistance of the cell was determined by impedance spectroscopy to be 10 Ω for the experiment performed at ID31, ESRF ,and 30 Ω for experiments performed at DanMAX, MAXIV. Before and after each potential step protocol, the reference potential of the Ag/AgCl electrode was measured against a freshly prepared RHE to ensure the stability of the reference electrode potential. All potentials are converted to the RHE scale. To continuously provide fresh electrolyte, the 0.5 M KOH ($KOH \cdot H_2O$, 99.995 % SupraPur® prepared from ultrapure water 18.2 MΩ MilliQ system) was pumped through the electrochemical cell with a constant flow rate of ca. 4 mL/min. At the ID31 beamline, a biologic SP-150 potentiostat was used, while a Biologic VMP3 potentiostat was used at the DanMAX beamline. Both potentiostats were using the Biologic EC lab control software.

Operando X-ray scattering data were collected in two different experiments at two different beamlines. XRD data and small angle X-ray scattering (SAXS) data were collected *quasi*-simultaneously at ID31 at the ESRF, Grenoble, France. The X-ray energy was 75 keV corresponding to an X-ray wavelength of λ = 0.165 Å. The beam size was 5 μm in the vertical direction with respect to the beam direction. The sample-to-detector distance was 800 mm for the XRD measurements and a *Q*-range from 0.1 to 14.3 $Å^{-1}$ was covered. For SAXS a sample-to-detector distance of 9987 mm covering a *Q*-range of 0.005 to 0.6 $Å^{-1}$. A Dectris Pilatus3 X CdTe 2M detector with 172×172 $μm^2$ pixel size, 1475×1679 pixel area was used to collect 2D scattering data which was subsequently azimuthally integrated with the PyFAI software.[3] The SAXS data were collected on a Perkin Elmer XRD 1621 detector with 200×200 $μm^2$ pixel size and a 2048×2048 pixel area.

Total scattering data for PDF analysis were collected at the DanMAX beamline at the MAXIV synchrotron in Lund, Sweden. Again, a Dectris Pilatus3 X CdTe 2M detector was used to collect 2D total scattering data, and PyFAI software to perform the azimuthal integration.[3] The beam size was 12μm in the vertical direction with respect to the beam direction. The sample-to-detector distance was 120.1 mm to cover a large *Q*-range, here 0.8 to 19.8 $Å^{-1}$, which allows a $Q_{maxinst}$ = 19.0 $Å^{-1}$, Q_{max} = 18.0 $Å^{-1}$, and Q_{min} = 1.4 $Å^{-1}$. The Q_{min} was chosen based on the position of the first reflection (003) in the data, which is interfering with the beam stop. Here, the X-ray energy was 35 keV corresponding to an X-ray wavelength of λ = 0.354 Å.

For the XRD, SAXS, and total scattering experiments, a similar electrochemical measurement protocol using chronoamperometry was employed. The protocol consisted of stepping the potential from 1.0 V_{RHE} to 1.6/1.7 V_{RHE}, in potential steps of 0.1 V. At each potential step, the potential was held for 2 min to reach *quasi-steady-state* conditions, before the scattering measurements were started. A height scan (z-scan) along the vertical to the beam direction (z-axis), was then performed through the GDL+MPL, catalyst layer, and electrolyte (KOH) for each potential step.

For the XRD measurements, data were collected with a time resolution of 1 s at 60 z-scans, resulting in 60 XRD patterns for each potential step.

For the PDF measurements, the time resolution of each scattering pattern was 10 s with 20 z-scans, resulting in 20 total scattering patterns for each potential step. In this way, we obtained scattering data of the electrochemical cell (PSU), the electrolyte, and the carbon support for appropriate background subtraction. The background subtraction was performed using a simple fitting procedure minimizing the difference between the measured sample (including background) and the linear combination of the different background contributions. An example of the background subtraction is presented in Figure S1 in the Supporting Information.

For the total scattering experiments conducted at the DanMAX beamline, the data were further treated to obtain the PDFs using the PDFgetX3 software.[4] The PDF modelling was performed using the PDFgui[5] and DiffPy-CMI programs.[6]

Rotating disk electrode (RDE) measurements

Catalyst inks were prepared by dispersing the dried catalyst powder in EtOH absolute (99.8 %, VWR) at a concentration of 0.5 mg_{sample} mL^{-1}. The ink was homogenized using a horn sonicator (Qsonica sonicator, Q500). While sonicating, the inks were cooled in an ice bath to prevent solvent evaporation. Glassy carbon RDE tips (5 mm diameter) were rinsed and sonicated in ultrapure water (Milli-Q IQ 7000, 18.2 MΩ cm, 2.7 ppb TOC). Then, they were mechanically polished on a polishing cloth (MicroCloth, Buehler) with an alumina slurry (0.3 µm, MicroPolish; Buehler) for 2 min, followed by rinsing and sonicating in ultrapure water. 19.6 µL of the prepared inks were pipetted onto the polished RDE tips, resulting in a catalyst loading of 50 µg cm^{-2}. The tips were dried under a humidified Ar-stream. The disks were mounted on an EDI101 rotating disk assembly (Radiometer Analytical).

The electrochemical measurements were conducted in a custom-built PTFE cell.[9] Before use, the cell was cleaned by boiling in 25% HNO_3, after which the cell was rinsed using ultrapure water and boiled therein. Ar-purged 0.1 M KOH (EMSURE, Merck) solution served as electrolyte. The prepared disks were used as WE, a Au wire as CE, and a saturated calomel electrode (SCE) as RE. The RE's shift versus the reversible hydrogen electrode was determined following the protocol outlined by Inaba et al.[10] In short, a polished Pt disk was cycled between −1.075 to 0.05 V_{SCE} at 0.050 V s^{-1} in H_2-saturated 0.1 M KOH versus the SCE. 0 V_{RHE} was determined to be where the hydrogen evolution and oxidation currents cancel each other out.

The measurements were controlled by an ECi 210 potentiostat (Nordic Electrochemistry ApS) via the EC4 DAQ 4.2 software (Nordic Electrochemistry, ApS). The software also controlled the applied convection through a CTV101 speed control unit (Radiometer Analytical).

The solution resistance was determined using a superimposed AC perturbation (5 mV amplitude at 5 kHz) and was compensated using an active feedback scheme to < 2 Ω. The remaining *iR* was corrected after the measurement.

Cyclic voltammograms were recorded between 1.2 V_{RHE} and 1.8 V_{RHE} at a scan rate of 0.5 mV s^{-1} and at a rotation speed of 2500 rpm to remove evolved oxygen from the catalytic interface.

Section 1: Data treatment.

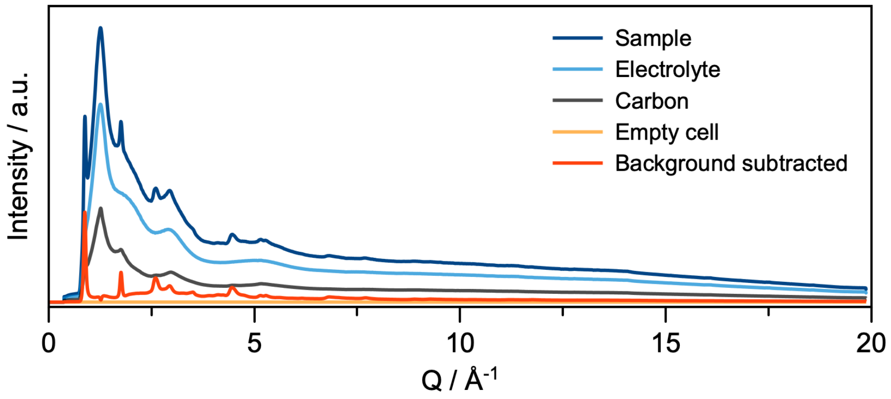

Figure S1. Scattering intensity from the electrolyte, carbon support, and the empty cell and their relative contribution to the background subtraction.

Section 2: Characterization of a-prepared catalyst material. The following figures and tables are related to analysis and refinements of the as-prepared material Ni4Fe:30h presented in the manuscript.

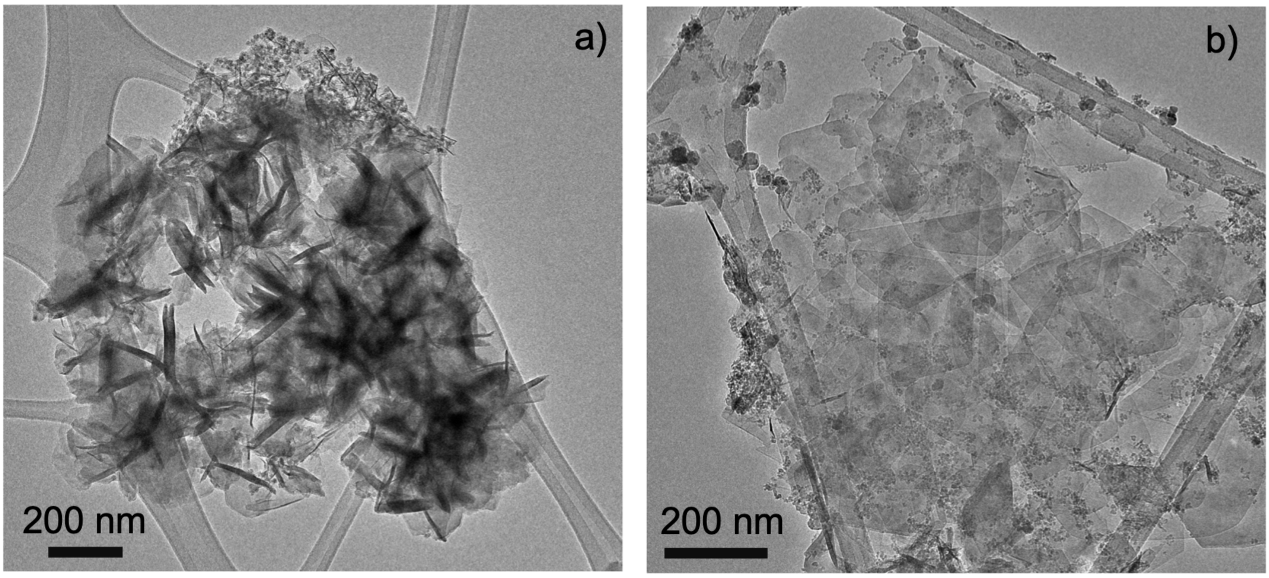

Figure S2. TEM micrographs of a) Ni4Fe:2h and b) Ni4Fe:24h. We note that the TEM image shown in Figure b) is for a sample prepared with 24h of synthesis, a slightly shorter synthesis time than the sample presented in the manuscript.

Table S1. Refined parameters for the PDF fits presented in Figure 2b and c in the manuscript. PDF from sample Ni4Fe:30h. α-LDH is used as the structural starting model with $U_{11} = U_{22} = U_{33}$ and with $U_{11} = U_{22} \neq U_{33}$. Parameters highlighted in grey were fixed in the refinement. For simplicity, we occupied all metal sites with Ni due to the similar X-ray scattering lengths of Fe and Ni. The ADP-values of O, C, and H sitting in between the layers were fixed to 0.003 Å^2.

	**α-LDH with isotropic ADPs**	**α-LDH with anisotropic ADPs**
	**Ni4Fe:30h**	**Ni4Fe:30h**
**Fit range**	1.7 – 60 Å	1.7 – 60 Å
**Number of refined parameters**	8	9
**R_w**	0.35	0.26
**Q_{damp} (Å^{-1})**	0.03	0.03
**Q_{broad} (Å^{-1})**	0.001	0.001
**Q_{max} (Å^{-1})**	20	20
**Scale factor**	0.34	0.33
**U_{aniso} for Ni (U_{11} and U_{22})**	0.0072	0.0038
**U_{aniso} for Ni (Å^2) (U_{33})**	= U_{11} and U_{22}	0.20
**U_{iso} for O (Å^2)**	0.017	0.013
**U_{iso} for C (intercalated) (Å^2)**	0.003	0.003
**U_{iso} for H (intercalated) (Å^2)**	0.003	0.003
**U_{iso} for O (intercalated) (Å^2) (O17-O23, O40-O46, O63-O69)**	0.003	0.003
**Lattice par., *a* (Å)**	12.4	12.4
**Lattice par., *b* (Å)**	6.21	6.17
**Lattice par., *c* (Å)**	23.6	23.5
**δ_2 (Å^2)**	2.81	3.74
**Sp-diameter (Å)**	35.4	54.5

Table S2. Fitting information from PDF fits shown in Figure 2c in the manuscript using a single sheet cut from the α-LDH with 28 Å radius as the structural starting model. Parameters highlighted in grey were fixed in the refinement.

	**Sheet with 28 Å radius from α-LDH**
	**Ni4Fe:30h**
**Fit range**	1.7 – 60 Å
**Number of refined parameters**	6
**R_w**	0.34
**Q_{damp} (Å^{-1})**	0.03
**Q_{broad} (Å^{-1})**	0.001
**Q_{max} (Å^{-1})**	20
**Scale factor**	0.45
**Zoomscale a**	0.992
**Zoomscale b**	1.00
**Zoomscale c**	0.983
**U_{iso} for Ni (Å^2)**	0.0065
**U_{iso} for O (Å^2)**	0.003
**δ_2 (Å^2)**	1.82

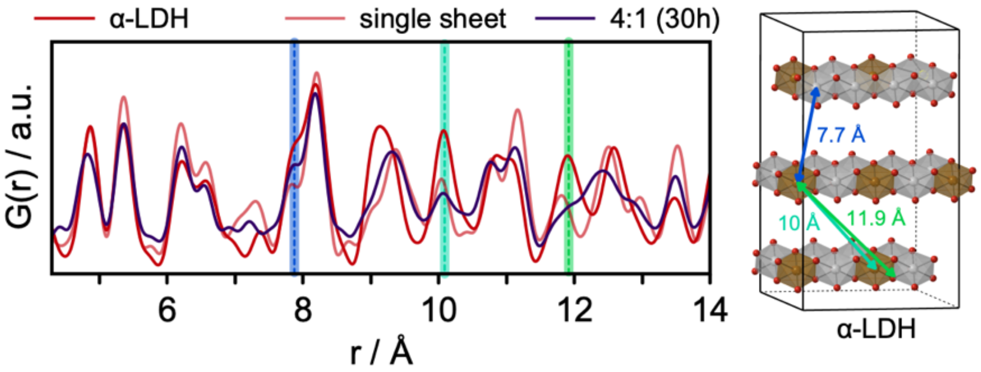

Figure S3. Comparing experimental PDFs with simulated PDFs from the α-LDH and a discrete single sheet. PDF peaks originating from atom-atom correlations between layers are highlighted and illustrated in the crystal structure.

Section 3: *Operando* XRD experiments. The following figures and tables are related to the refinements and analysis performed for the *operando* XRD experiments presented in the manuscript.

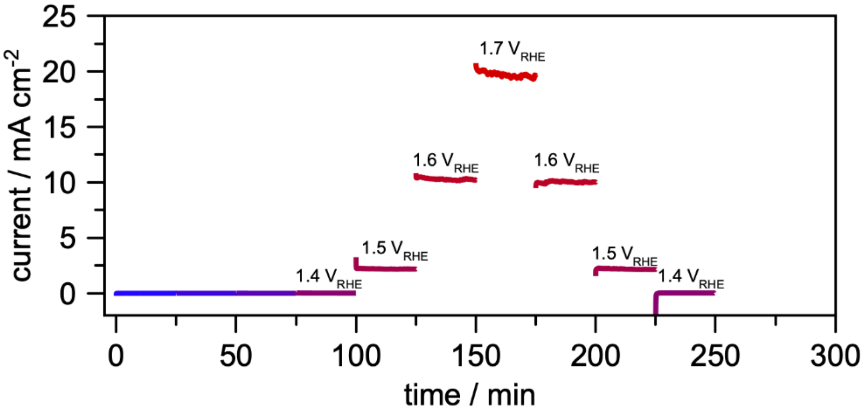

Figure S4. Chronoamperometry for the sample Ni4Fe:30h collected during OER in the *operando* electrochemical cell, related to the XRD data collected and presented in Figure 3b and c.

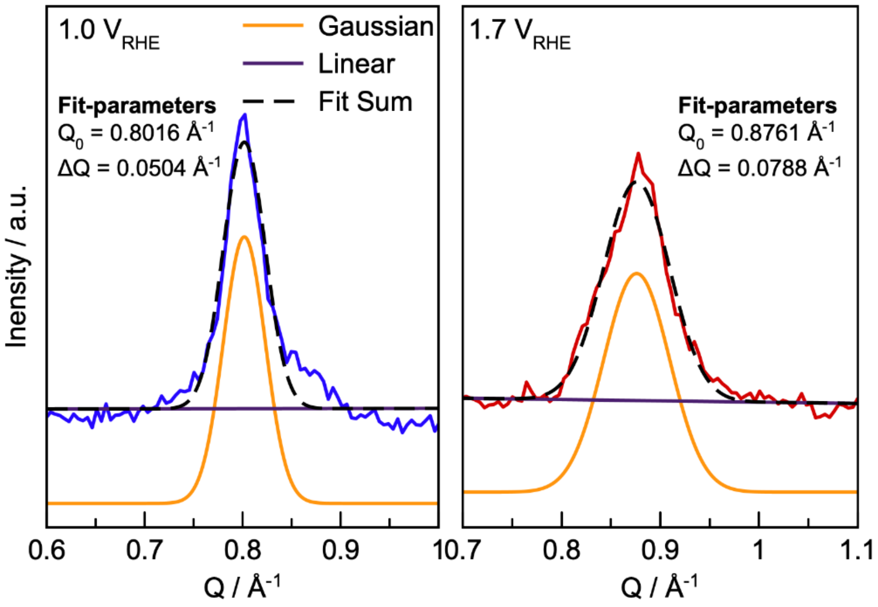

Figure S5. Examples of the Gaussian fitting for two selected XRD patterns. The fittings are used for extracting the Q-value of the maximum height, which is then used to extract the d-spacing using Bragg's law. The ΔQ is used to investigate the peak width. Figure 3d and Figure S5 summarize the results from the Gaussian fitting of the whole XRD series.

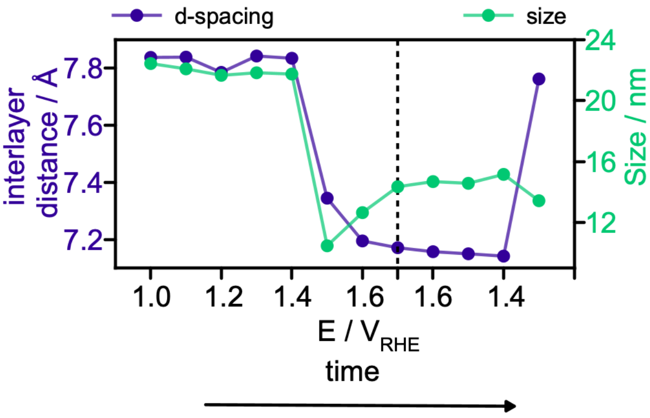

Figure S6. *d*-spacing and estimated crystallite size as a function of applied potential. The size is estimated using the (003) peak width (FWHM) in the Scherrer equation. Here, all the peak broadening is attributed to the size effect, excluding any instrumental broadening.

Section 4: *Operando* scattering experiments. The following figures and tables are related to the refinements and analysis performed for the *operando* total scattering experiments presented in the manuscript.

The cell used for all operando X-ray scattering experiments (total scattering, XRD, and SAXS) has been reported by Wiberg et al.[7] . and details of the cell design are described therein. A picture of the cell installed at the ID31 beamline at the ESRF is shown on the left in Figure S7. Here the beam path through the cell and the position of the cell with respect to the detector is shown. All electrode connections as well as the tubing for the electrolyte flow in and out of the cell are highlighted. The electrolyte is flown through the cell by a two-channel peristaltic pump.
The electrochemical response of the catalyst studied at both the ESRF and DanMAX is comparable, as visible in the Tafel plot in Figure S7. The potentials are recalculated for uncompensated resistance and therefore differ slightly. This is a result of variations in cell geometries due to using two different reference electrodes. While we used a large RHE in the experiments at DanMAX, a much thinner Ag|AgCl reference electrode (eDAQ) was used at the ESRF. The distance between WE and RE was shorter in the case of the Ag|AgCl RE. This decreased the solution resistance in the experiments and the effective applied potential at the ESRF was, therefore, higher. A picture of the bubble formation in the cell is presented in Figure S7 (right).

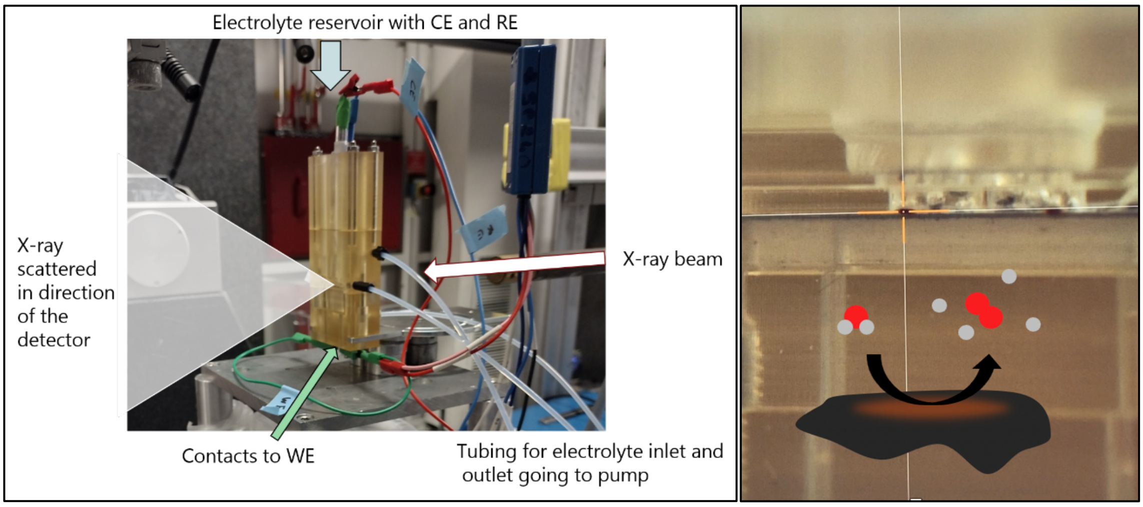

Figure S7. Left: Image of the *operando* cell for X-ray scattering mounted at the ID31 beamline, highlighting the beam path, electrode connections, and electrolyte tubing. Right: Microscopy image showing the bubble evolution inside the cell at an applied potential of 1.7 V vs RHE where significant oxygen evolution activity occurs.

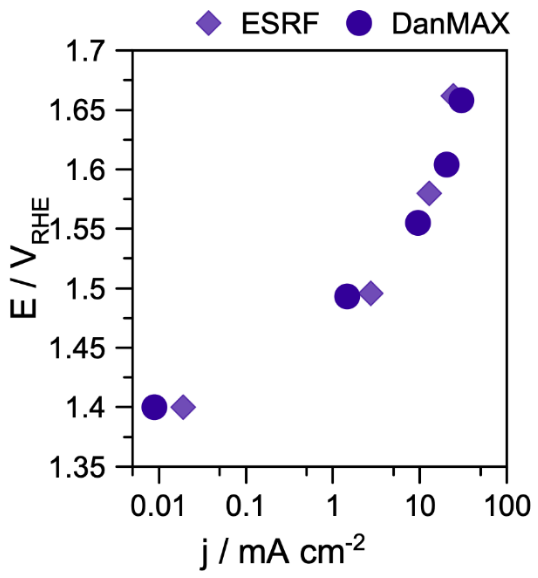

Figure S8. Oxygen evolution Tafel plots Ni4Fe:30h derived from the steady-state measurements showing the recorded current densities after applying the potential for 10 minutes comparing the electrochemistry from the two experiments at the two different beamlines (ID31 and DanMAX).

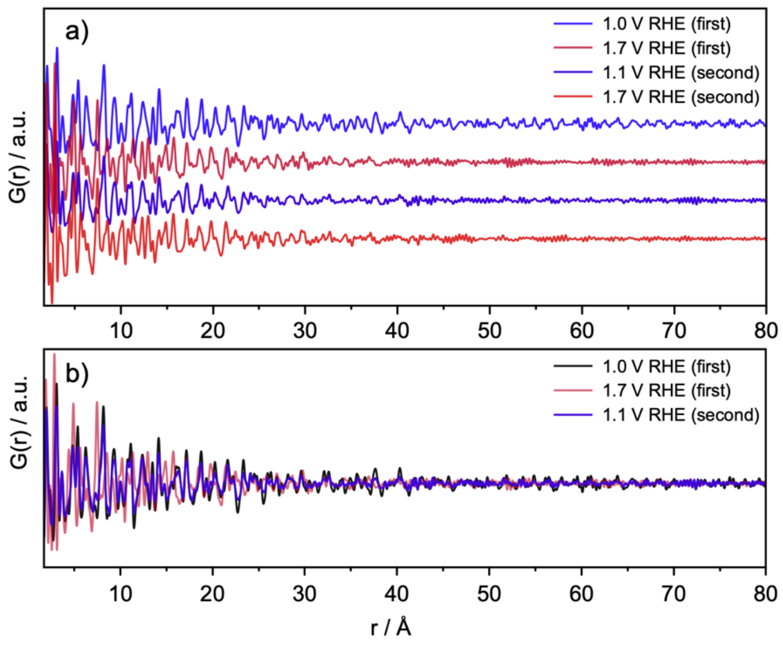

Figure S9. Selected PDFs from Figure 4 (sample Ni4Fe:30h) in the manuscript are illustrated for direct comparison of the dampening effect.

Table S3. *Operando* total scattering and PDF refined parameters. PDF refinements for the sample Ni4Fe:30h using the α-LDH or γ-LDH as the structural starting model. Anisotropic ADP values are used in all refinements. Parameters highlighted in grey were fixed in the refinement.

	**Ni4Fe:30h**			
	**α-LDH**	**γ-LDH**	**α-LDH**	**γ-LDH**
**Potential**	**1.0 V_{RHE} (before OER)**	**1.7 V_{RHE} (first)**	**1.1 V_{RHE} (after OER)**	**1.7 V_{RHE} (second)**
**Fit range**	1.7 – 60 Å	1.7 – 60 Å	1.7 – 60 Å	1.7 – 60 Å
**Number of refined parameters**	9	9	9	9
**R_w**	0.41	0.51	0.47	0.46
**Q_{damp} (Å^{-1})**	0.021	0.021	0.021	0.021
**Q_{max} (Å^{-1})**	18.0	18.0	18.0	18.0
**Scale factor**	0.41	0.27	0.31	0.32
**U_{aniso} for Ni (Å^2) (U_{11} and U_{22})**	0.0054	0.0014	0.0085	0.0003
**U_{aniso} for Ni (Å^2) (U_{33})**	0.14	0.17	0.11	0.46
**U_{iso} for C (intercalated) (Å^2)**	0.003	0.003	0.003	0.003
**U_{iso} for H (intercalated) (Å^2)**	0.003	0.003	0.003	0.003
**U_{iso} for O (intercalated) (Å^2) (O17-O23, O40-O46, O63-O69)**	0.003		0.003	
**U_{iso} for O (intercalated) (Å^2) (O17-O20)**		0.003		0.003
**U_{iso} for K (intercalated) (Å^2)**		0.003		0.003
**U_{iso} for O (Å^2)**	0.011	0.015	0.012	0.009
**Lattice par., *a* (Å)**	12.4	9.85	12.4	11.4
**Lattice par., *b* (Å)**	6.19	5.65	6.19	5.64
**Lattice par., *c* (Å)**	23.5	7.30	23.6	22.2
**δ_2 (Å^2)**	3.25	3.54	2.57	3.52
**Sp-diameter (Å)**	66.9	31.4	39.1	38.6

Table S4. PDF refinements for the sample Ni4Fe:30h using the sheets cut out from the α-LDH as the structural starting model. The sheet radius was determined from several refinements with sheets ranging from 10 to 65 Å in radius. The model resulting in the lowest R_w was determined to be the most suitable sheet size. Parameters highlighted in grey were fixed in the refinement.

We observed that the *a*- and *b*-values used for zoom-scaling are both decreasing during the first and second time of applied OER potential (1.6 V_{RHE}). This trend agrees with the finding from the model-free analysis presented in Figure 4c-d, where we observe this contraction in the very local *r*-range in the *a*/*b*-plane, related to the Ni oxidation.

**Ni4Fe:30h**				
**Single sheets from α-LDH**				
**Potential**	**1.0 V_{RHE} (before OER)**	**1.7 V_{RHE} (first)**	**1.1 V_{RHE} (after OER)**	**1.7 V_{RHE} (second)**
**Sheet radius(Å)**	38	26	28	24
**Q_{damp} (Å^{-1})**	0.021	0.021	0.021	0.021
**Q_{max} (Å^{-1})**	18.0	18.0	18.0	18.0
**R_w**	0.43	0.44	0.43	0.41
**Scale**	0.58	0.33	0.42	0.41
**Zoomscale a**	1.0	0.91	1.00	0.92
**Zoomscale b**	0.99	0.91	0.99	0.91
**Zoomscale c**	0.93	0.98	0.88	0.98
**δ_2 (Å^2)**	0.91	5.0	1.6	5.3
**U_{iso} for Ni (Å^2)**	0.0089	0.0082	0.013	0.0083
**U_{iso} for O (Å^2)**	0.003	0.003	0.003	0.003

Section 5: *Operando* SAXS experiments. The following figures and tables are related to the analysis performed for the *operando* SAXS experiments presented in the manuscript.

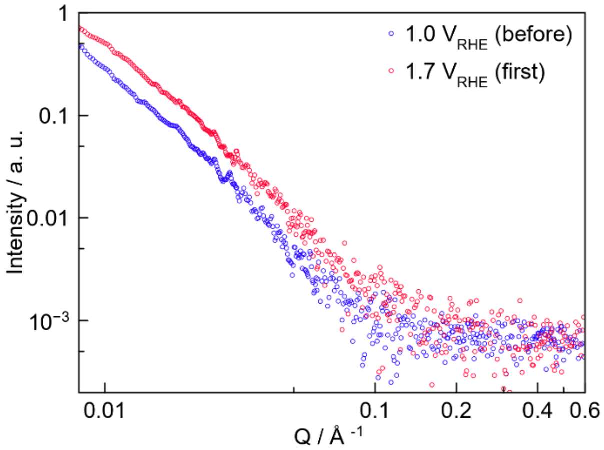

Figure S10. SAXS collected at 1.0 and 1.7 V_{RHE}. The scattering data have been corrected for background scattering from the cell and the Carbon support.

Table S5. Parameters of the SAXS fit for the sample Ni4Fe:30h at different potentials with a polydisperse spherical model and a power law. The underlying size distribution was chosen to be lognormal. The fits were done with SasView 5.0.6. The fit range was from 0.008 to 0.2 $Å^{-1}$.

Ni4Fe:30h		
**Potential**	**1.0 V_{RHE} (before)**	**1.7 V_{RHE} (first)**
**Mean Radius (Å)**	46	39
**Polydispersity**	0.45	0.45
**Scale Sphere**	0.00046	0.0006
**Power**	4	2.8
**Scale Powerlaw**	1.5E-9	1.0E-6
**Background (cm^{-1})**	0.0008	0.0009

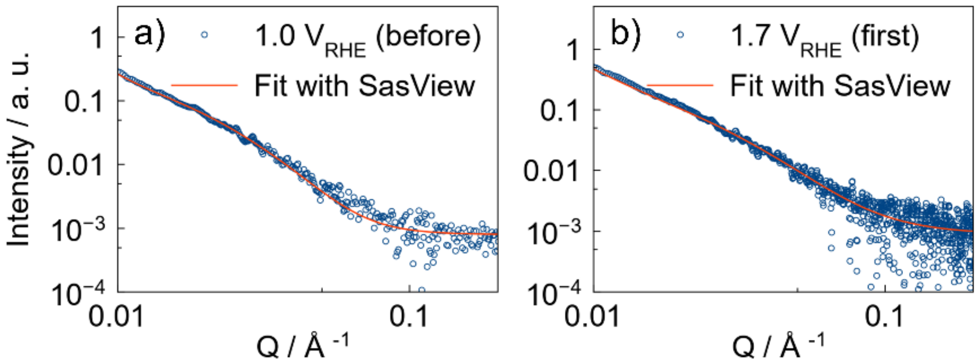

Figure S11. Fits with a powerlaw and a polydisperse sphere of the SAXS data with SasView. An overview of the fitting parameters is presented in Table S5.

Section 6: *Operando* XRD and PDF on additional samples. The following figures and tables are related to the refinements and analysis performed for the *operando* total scattering experiments presented in the manuscript.

Table S6. Refined parameters for the PDF fits presented in Figure S12. PDF from samples **Ni4Fe:2h and Ni:2h.** α-LDH is used as the structural starting model with $U_{11} = U_{22} = U_{33}$ and with $U_{11} = U_{22} \neq U_{33}$. Parameters highlighted in grey were fixed in the refinement.

	**α-LDH with isotropic ADPs**	**α-LDH with anisotropic ADPs**	**α-LDH with isotropic ADPs**	**α-LDH with anisotropic ADPs**
	**Ni4Fe:2h**	**Ni4Fe:2h**	**Ni:2h**	**Ni:2h**
**Fit range**	1.7 – 60 Å	1.7 – 60 Å	1.7 – 60 Å	1.7 – 60 Å
**Number of refined parameters**	8	9	8	9
**R_w**	0.57	0.30	0.68	0.42
**Q_{damp} (Å^{-1})**	0.03	0.03	0.021	0.021
**Q_{broad} (Å^{-1})**	0.001	0.001	0.001	0.001
**Q_{max} (Å^{-1})**	20	20	18	18
**Scale factor**	0.35	0.33	0.28	0.28
**U_{aniso} for Ni (U_{11} and U_{22})**	0.0035	0.0051	0.0050	0.0067
**U_{aniso} for Ni (Å^2) (U_{33})**	= U_{22}	1.00	= U_{22}	2.76
**U_{iso} for O (Å^2)**	0.028	0.011	0.035	0.021
**U_{iso} for C (intercalated) (Å^2)**	0.003	0.003	0.003	0.003
**U_{iso} for H (intercalated) (Å^2)**	0.003	0.003	0.003	0.003
**U_{iso} for O (intercalated) (Å^2) (O17-O23, O40-O46, O63-O69)**	0.003	0.003	0.003	0.003
**Lattice par., *a* (Å)**	12.45	12.47	12.48	12.50
**Lattice par., *b* (Å)**	6.193	6.211	6.237	6.236
**Lattice par., *c* (Å)**	21.01	22.16	20.34	20.28
**δ_2 (Å^2)**	2.80	4.05	2.26	2.76
**Sp-diameter (Å)**	24.0	55.1	25.6	91.4

Table S7. Refined parameters from PDF fits shown in Figure S12 using a single sheet cut from the α-LDH as the structural starting model. PDF from samples **Ni4Fe:2h** (Figure S12d) **and Ni:2h** (Figure S12h)**.** Parameters highlighted in grey were fixed in the refinement.

	**Sheet with 34 Å radius from α-LDH**	**Sheet with 65 Å radius from α-LDH**
	**Ni4Fe:2h**	**Ni:2h**
**Fit range**	1.7 – 60 Å	1.7 – 60 Å
**Number of refined parameters**	6	6
**R_w**	0.30	0.40
**Q_{damp} (Å^{-1})**	0.03	0.021
**Q_{broad} (Å^{-1})**	0.001	0.001
**Q_{max} (Å^{-1})**	20	18
**Scale factor**	0.40	0.35
**Zoomscale a**	1.010	1.000
**Zoomscale b**	0.998	1.010
**Zoomscale c**	0.970	0.951
**U_{iso} for Ni (Å^2)**	0.0066	0.0084
**U_{iso} for O (Å^2)**	0.003	0.003
**δ_2 (Å^2)**	1.7	0.099

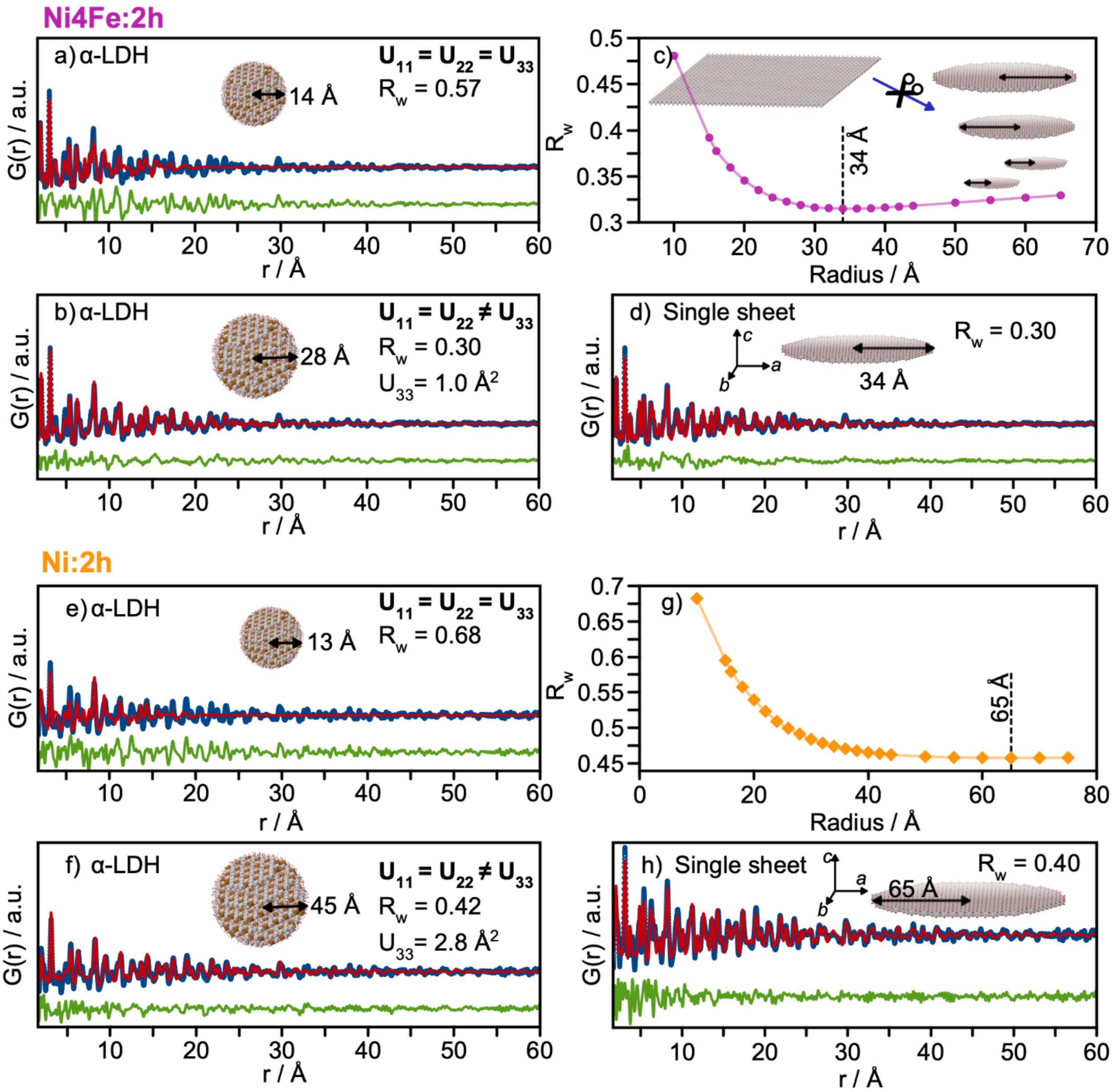

Figure S12. PDF refinements for the PDFs of the as-prepared material: **Ni4Fe:2h and Ni:2h** using both α-LDH and the sheet models.

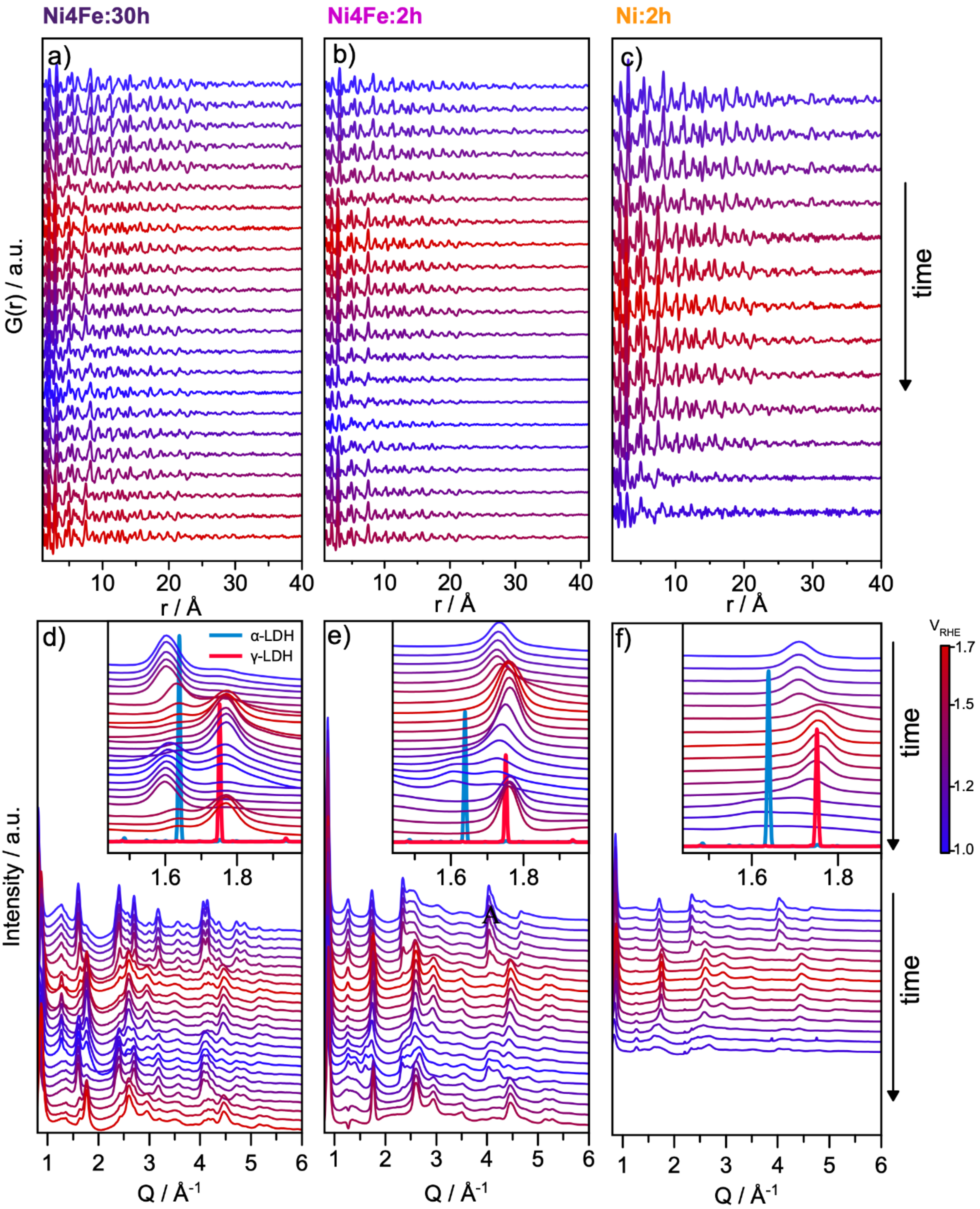

Figure S13. PDFs from the *operando* total scattering data of the three samples. a) Ni4Fe:30h b) Ni4Fe:2h, and c) Ni:2h and the respective total scattering data in d), e) and f), respectively, following a potential step protocol with applied potential of 1.0 V_{RHE} to 1.7 V_{RHE} with steps of 0.1 V_{RHE}. The (006) reflections are followed in the inserts.

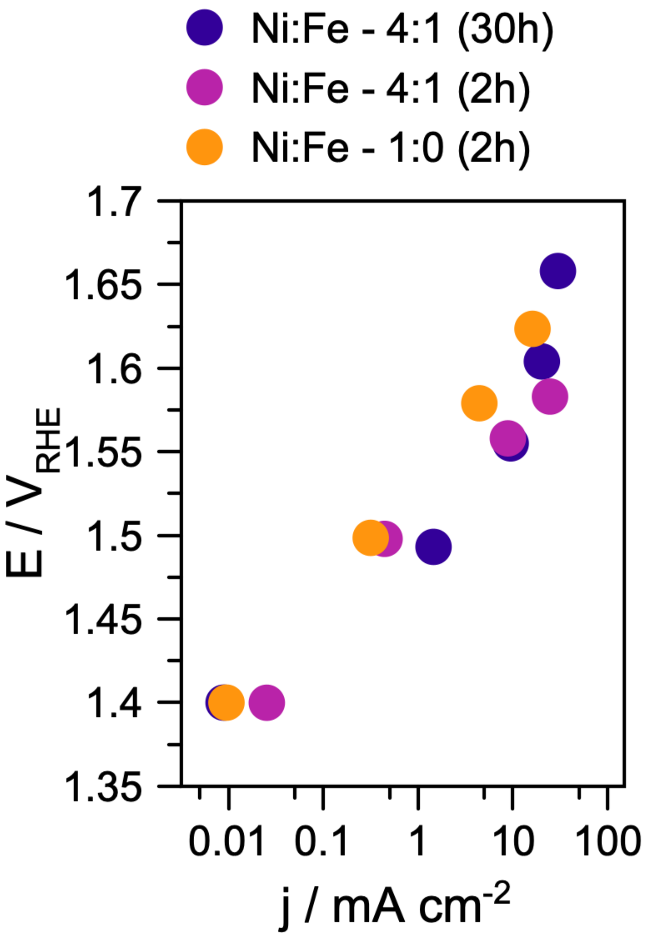

Figure S14. Oxygen evolution Tafel plots of Ni4Fe:30h, Ni4Fe:2h, and Ni:2h derived from the steady-state measurements showing the recorded current densities after applying the potential for 10 minutes.

References

1. Lu, Z.; Xu, W.; Zhu, W.; Yang, Q.; Lei, X.; Liu, J.; Li, Y.; Sun, X.; Duan, X., Three-dimensional NiFe layered double hydroxide film for high-efficiency oxygen evolution reaction. *Chem. Commun.* **2014,** *50* (49), 6479-6482.
2. Wang, B.; Schlegel, N.; Aalling-Frederiksen, O.; Berner, E.; Zhang, D.; Pittkowski, R. K.; Jensen, K. M.; Arenz, M., (Ni1–x Fe x) OOH Binary Nanoparticles in as-Prepared and Purified KOH Electrolyte Solutions for Water Splitting. *ACS Appl. Nano Mater.* **2024**.
3. Kieffer, J.; Karkoulis, D. In *PyFAI, a versatile library for azimuthal regrouping*, J. Phys. Conf. Ser., IOP Publishing: 2013; p 202012.
4. Juhás, P.; Davis, T.; Farrow, C. L.; Billinge, S. J., PDFgetX3: a rapid and highly automatable program for processing powder diffraction data into total scattering pair distribution functions. *J. Appl. Crystallogr.* **2013,** *46* (2), 560-566.
5. Farrow, C.; Juhás, P.; Liu, J.; Bryndin, D.; Božin, E.; Bloch, J.; Proffen, T.; Billinge, S., PDFfit2 and PDFgui: computer programs for studying nanostructure in crystals. *Journal of Physics: Condensed Matter* **2007,** *19* (33), 335219.
6. Juhás, P.; Farrow, C.; Yang, X.; Knox, K.; Billinge, S., Complex modeling: a strategy and software program for combining multiple information sources to solve ill posed structure and nanostructure inverse problems. *Acta Crystallogr. A* **2015,** *71* (6), 562-568.
7. Wiberg, G. K. H.; Pittkowski, R. K.; Punke, S.; Aalling-Frederiksen, O.; Jensen, K. M. Ø.; Arenz, M., Design and Application of a Gas Diffusion Electrode (GDE) Cell for Operando and In Situ Studies. *Chimia* **2024**.
8. Wiberg, G. K.; Nösberger, S.; Arenz, M., Evolution of a GDE setup: Beyond ambient conditions. *Current Opinion in Electrochemistry* **2022,** *36*, 101129.
9. Mayrhofer, K.; Wiberg, G.; Arenz, M., Impact of glass corrosion on the electrocatalysis on Pt electrodes in alkaline electrolyte. *J. Electrochem. Soc.* **2007,** *155* (1), P1.
10. Inaba, M.; Quinson, J.; Bucher, J. R.; Arenz, M., On the Preparation and Testing of Fuel Cell Catalysts Using the Thin Film Rotating Disk Electrode Method. *JoVE* **2018,** (133), e57105.
